# The Added Value of Medical Testing in Underwriting Life Insurance

**DOI:** 10.1371/journal.pone.0145891

**Published:** 2015-12-30

**Authors:** Jan Bronsema, Sandra Brouwer, Michiel R. de Boer, Johan W. Groothoff

**Affiliations:** 1 Legal & General, Hilversum, The Netherlands; 2 Department of Health Sciences, Division of Community and Occupational Medicine, Groningen, University Medical Center Groningen, University of Groningen, Groningen, The Netherlands; 3 Academic Center Private Insurance Medicine, Groningen, The Netherlands; 4 Department of Health Sciences, VU University, Amsterdam, The Netherlands; University of California, Berkeley, UNITED STATES

## Abstract

**Background:**

In present-day life-insurance medical underwriting practice the risk assessment starts with a standard health declaration (SHD). Indication for additional medical screening depends predominantly on age and amount of insured capital. From a medical perspective it is questionable whether there is an association between the level of insured capital and medical risk in terms of mortality. The aim of the study is to examine the prognostic value of parameters from the health declaration and application form on extra mortality based on results from additional medical testing.

**Methods:**

A history register-based cohort study was conducted including about 15.000 application files accepted between 2007 and 2010. Blood pressure, lipids, cotinine and glucose levels were used as dependent variables in logistic regression models. Resampling validation was applied using 250 bootstrap samples to calculate area under the curves (AUC’s). The AUC was used to discriminate between persons with and without at least 25% extra mortality.

**Results:**

BMI and the overall assessment of the health declaration by an insurance physician or medical underwriter showed the strongest discrimination in multivariable analysis. Including all variables at minimum cut-off levels resulted in an AUC of 0.710 while by using a model with BMI, the assessment of the health declaration and gender, the AUC was 0.708. Including all variables at maximum cut-off levels lead to an AUC of 0.743 while a model with BMI, the assessment of the health declaration and age resulted in an AUC of 0.741.

**Conclusions:**

The outcome of this study shows that BMI and the overall assessment of the health declaration were the dominant variables to discriminate between applicants for life-insurance with and without at least 25 percent extra mortality. The variable insured capital set by insurers as factor for additional medical testing could not be established in this study population. The indication for additional medical testing at underwriting life-insurance can possibly be done on limited variables instead of the obligatory medical testing based on age and the amount of insured capital.

## Introduction

The added value of screening is a constant debate in medicine [1]. Screening as an instrument in insurance medicine is no exception to this [2], particularly not in the context of the accessibility to private insurances [3]. For underwriting life-insurance, insurance companies assess the mortality risk of applicants. The level of medical guarantees to be delivered by the applicant predominantly depends on age and the amount of insured capital [4]. Guarantees range from a standard health declaration (SHD) to a SHD accompanied by a medical examination, including laboratory testing.

In present-day life-insurance underwriting practice, the SHD is the starting-point in the risk assessment. Depending on the amount of insured capital and age additional medical examination, including standard laboratory tests like lipids and glucose, will be the obligatory required guarantee, regardless of the outcome of the health declaration. The validity of age as an indicator for the extent of medical guarantees is undisputable. Higher age is associated with higher mortality rates [5,6,7]. The amount of insured capital as indicator for the level of medical guarantees is set by the insurer. The higher the financial risk in terms of insured capital the more guarantees the insurer will ask from the applicant [8]. From a medical perspective it is questionable whether there is an association between the level of insured capital and medical risk in terms of mortality. If the level of amount insured can be interpreted as a derivative of the socio-economic standard and related health behavior of the applicant there could even be an inverse association with the mortality risk [9,10].

Medical examinations are costly, bothersome for the applicant and time-consuming in the underwriting process [8]. This raises the question whether it is possible to predict the probability of finding a relevant extra-mortality due to blood pressure and/or lipid or glucose levels, given the information from the SHD. Whether screening for these risk factors in a relative healthy population applying for life-insurance makes sense in terms of the chance of finding an increased mortality risk is hardly known as evidence-based ratings in life insurance are predominantly based on existing morbidity in clinical literature [11]. Therefore the aim of this study is to examine the prognostic value of parameters from the health declaration and application form in a sample of life-insurance applicants on extra mortality risks based on the outcome of additional medical testing. An answer may lead to a more focused and efficient medical underwriting procedure.

## Methods

### Design and procedure

The Dutch Data Protection Agency (DPA) and the Code of Conduct of Medical Officers in the Netherlands allows insurance companies to utilize anonymized data for scientific purposes.

For this historic register-based cohort study we used data of consecutive applications for life-insurances at one of the life-insurance companies in the Netherlands between 2007 and 2010. The data were collected after the introduction of a ‘preferred life’ product to the existing product range. ‘Preferred life’ insurance has reduced premium rates compared to standard rates for applicants fulfilling strict medical criteria, expecting a lower mortality than their age-standardized fellow insured [12]. Files regarding the second or more application of the same applicant were excluded from analyses, as were files for which medical testing data were not available. Medical data were not available because of administrative errors or were not registered if applicants did not meet with observable technical and/or financial criteria like sum assured. Only data of obligatory additional medical testing were registered. Type of cover was limited to the standard term life insurance: cover guaranteed for limited period of time.

### Independent variables

Non-medical data were retrieved from the application form. Data included the amount of insured capital, whether the applicant applied for a ‘preferred life’ insurance policy and whether the applicant belonged to higher socio-economic standard in terms of income or asset. Amount of insured capital was categorized into five classes ranging from ≤ €250.000 to > €1.500.000. Preferred life rate was applicable if the applicant fulfilled strict criteria at medical examination. The criteria are shown S1 Table. A higher socio-economic standard was assumed in case the applicant had an income of ≥ 50.000 euro or assets of ≥ 200.000 euro, checked by bank account, salary strip or accountant declaration. Data retrieved from the health declaration were age, gender, family-history of first degree relatives, smoking history and the overall assessment of the health declaration by an insurance physician or medical underwriter whether there was an estimated extra-mortality of at least 25%. Finally, BMI as continuous variable was calculated by manually measured length and weight measured by digital weighing scales. See Table 1 for an overview of the independent variables.

**Table 1:** Variables and characteristics of the study sample. I = Independent variable; D = Dependent variable; SHD = standard health declaration; AF = application form; SD = standard deviation; ME = medical examination. #1: Non-smoker: signed declaration of not smoking or using nicotine containing products in the past 24 months and negative urine cotinine test. #2: The applicant assumes meeting the criteria set for reduced premium rates linked to 'Preferred Life' insurance. #3: Criteria are income of ≥ € 50.000 or asset of ≥ € 200.000. #4: Positive if estimated extra-mortality of at least +25% due to any disclosed medical condition and/or use of blood pressure or lipids lowering drugs. #5: First degree relatives under age 50 with diabetes, cancer or cardiovascular disease, including cerebrovascular accident.

Variable	I/D	Source	N	Descriptives
Gender	I	SHD	15.094	11.531 (76.4%) male
Age	I	SHD	15.094	Mean 40.2 yr (SD 6.5 yr)
Non-smoking (#1)	I	SHD/AF	15.094	11.185 (74.1%)
Insured capital	I	AF	15.094	Mean €430.193 (SD €355.493) Categories: ≤ €250.000 (28.9%), > €250.000 and ≤ €500.000 (50.3%), > €500.000 and ≤ €750.000 (11.1%), > €750.000 and ≤ €1.000.000 (6.0%), > €1.000.000 and ≤ €1.500.000 (2.4%), > €1.500.000 (1.3%)
Preferred Life application (#2)	I	AF	15.094	11.094 (73.5%)
Life-style application (#3)	I	AF	15.094	14.354 (95.1%)
Positive health declaration (#4)	I	SHD	15.094	5.539 (36.7%)
Positive family history (#5)	I	SHD	15.094	6.007 (39.8%)
BMI	I	ME	15.062	Mean 24.9 (SD 3.1)
Systolic blood pressure	D	ME	15.002	Mean 123 mmHg (SD 12 mmHg)
Diastolic blood pressure	D	ME	15.001	Mean 78 mmHg (SD 8 mmHg)
Total-cholesterol	D	ME	15.017	Mean 5.4 mmol/l (SD 1.1 mmol/l)
HDL-cholesterol		ME	14.969	Mean 1.4 mmol/l (SD 0.4 mmol/l)
Ratio chol/HDL	D	ME	14.969	Mean 4.0 (SD 1.2)
Triglycerides	D	ME	14.905	Mean 1.6 mmol/l (SD 1.0 mmol/l)
Glucose	D	ME	14.922	Mean 5.0 mmol/l (SD 0.9 mmol/l)
Cotinine	D	ME	14.856	Mean 18.5 ng/ml (SD 61.1 ng/ml)

### Dependent variables

From the additional medical testing data, we used systolic and diastolic blood pressure, level of total-cholesterol, the ratio of total-cholesterol and HDL-cholesterol, level of triglycerides and cotinine. Laboratory tests were all assessed in one laboratory. Cotinine was tested in case the applicant declared not to smoke or to use any nicotine containing products for the past 24 months. Cotinine was tested in urine. The medical assessment agencies measured blood pressure by digital electronic oscillometric devices.

**Fig 1:**
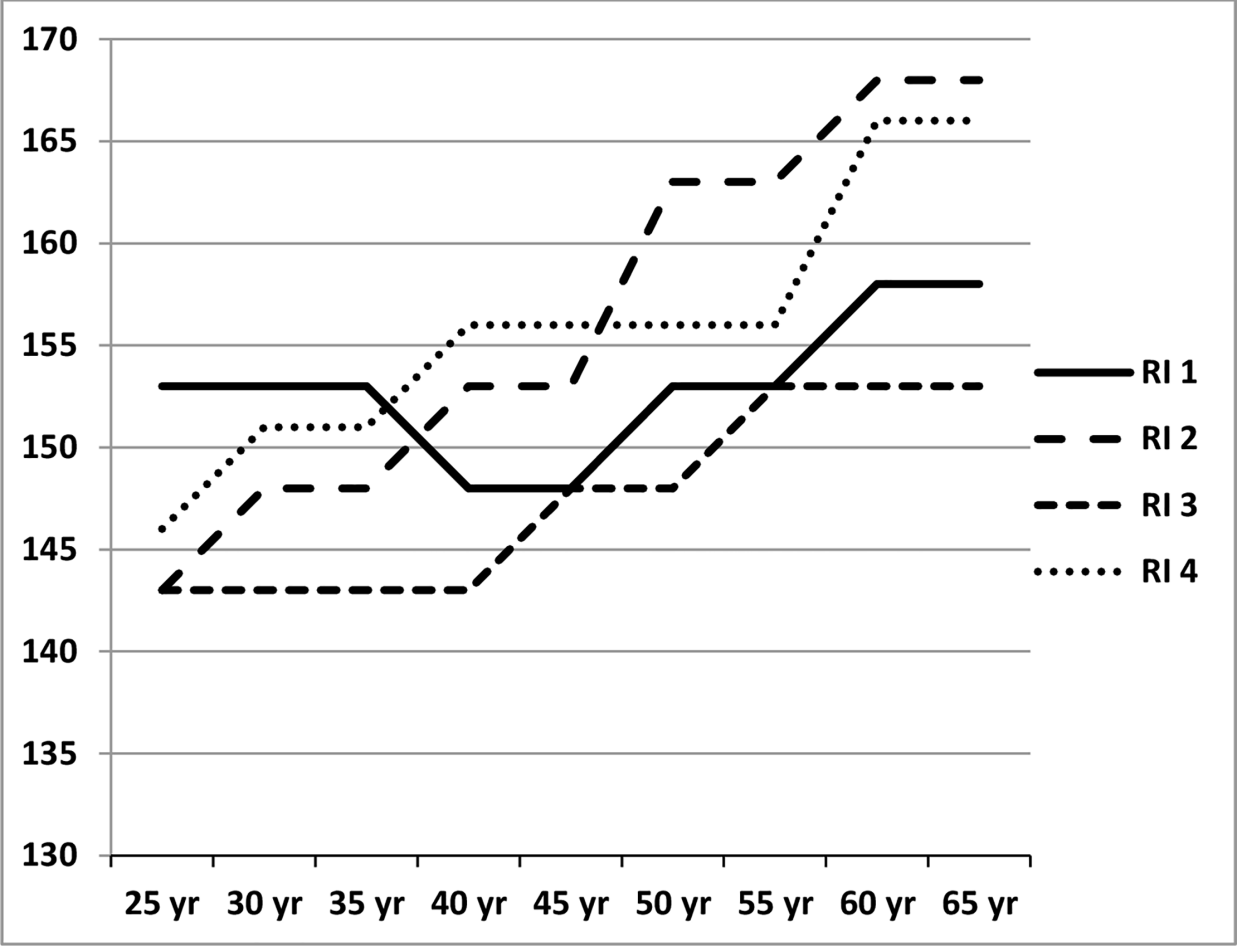
Cut-off values minimum loading +25% extra-mortality of four global reinsurer manuals (RI 1 to 4) for systolic blood pressure in mmHg: male/female. Data march 2012. RI 1 = cut-off values obtained from manual reinsurer 1. RI 2 = cut-off values obtained from manual reinsurer 2. RI 3 = cut-off values obtained from manual reinsurer 3. RI 4 = cut-off values obtained from manual reinsurer 4.

**Fig 2:**
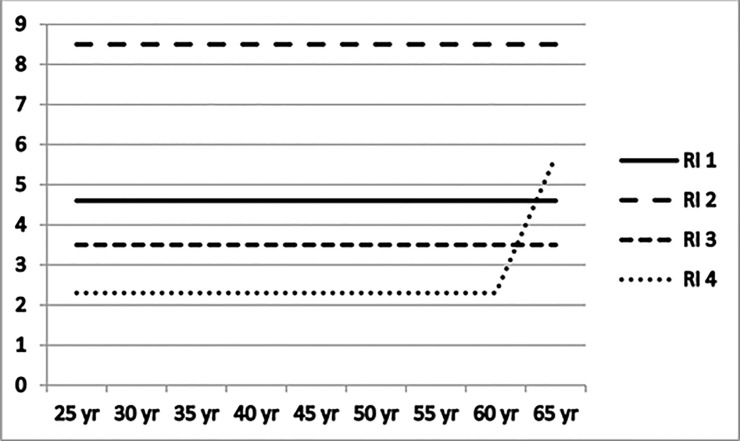
Cut-off values minimum loading +25% extra-mortality of four global reinsurer manuals (RI 1 to 4) for triglycerides in mmol/l: male. Data march 2012. RI 1 = cut-off values obtained from manual reinsurer 1. RI 2 = cut-off values obtained from manual reinsurer 2. RI 3 = cut-off values obtained from manual reinsurer 3. RI 4 = cut-off values obtained from manual reinsurer 4.

As a cut-off value for systolic and diastolic blood pressure, total-cholesterol, ratio of total-cholesterol and HDL-cholesterol and triglycerides, we used 25% extra-mortality, the minimum for an extra premium in life-insurance. The individual risk assessment of extra-mortality for every dependent variable from the medical testing, was based on the guidelines of four global reinsurance manuals. Examples of cut-off values for systolic blood pressure and triglyceride are shown in Figs 1 and 2. As the manuals show significant differences in the cut-off levels, the calculations were done on the overall minimum and maximum levels.

For the cut-off value for glucose the manuals follow the international acknowledged cut-off level of random glucose ≥ 7,8 mmol/l [13]. In case of glucose of ≥ 7,8 mmol/l an increased mortality risk of at least 25% is assumed based a glucose metabolism disorder. For the urine cotinine test the cut-off value of ≤ 25 ng/ml for non-smoking was applied [14]. In case of a positive cotinine test an elevated mortality risk is assumed based on the use of nicotine containing products. In practice premium rates for smokers will be applied instead of non-smokers premium rates. Premium rates for smokers are based on an extra mortality risk of more than 25% compared to non-smokers. For the ratio of total-cholesterol/HDL-cholesterol the cut-off values were retrieved from clinical literature: > 6.4 for men and > 5.6 for women [15].

An overall dependent variable was composed of all dependent variables except cotinine and defined positive in case of at least 25% extra-mortality due to one or more of the dependent medical variables.

### Statistical analysis

In order to investigate possible selection bias we compared the persons with and without medical data on age, gender, insured amount and whether the applicant belonged to higher socio-economic standard in terms of income or assets using chi-square tests or t-tests (income/assets/insured sum). Next, we examined which of the independent variables were significantly associated to our dependent variables.

For each dependent variable first univariable and then multivariable logistic regression analyses were performed. First, forward modeling in multivariable analysis was applied for the overall dependent variable. Subsequently the same order of inclusions was used for all separate models. We performed resampling validation by using 250 bootstrap samples and present the corrected (shrunken for overfitting) area under the curves (AUC’s). The AUC is a measure of the discriminative ability of a model. In this case that means that the higher the AUC, the better the (independent variables in the) model are able to discriminate between persons with and without at least 25% extra mortality based on the dependent variables from the medical examination. Analysis were done for both the minimum and the maximum cut-off levels for blood pressure and lipids. Additional calculation on the AUC were done using models including only the variables with significant impact on the predicted probability in the multivariable logistic regression analyses. The statistical analyses were performed using SPSS-21 software and R 3.2.2., package lrm for the resampling validation. The significance level was set at < .05.

## Results

### Missing data analyses

The insurance company received 20.966 consecutive applications for life insurances in the inclusion period. After excluding files regarding the second or more application of the same applicant, 19.143 files remained. For 4107 persons the additional medical testing data were not available and these persons were therefore excluded from the analyses. These persons differed from the persons of whom medical testing data was available with respect to age (41.0 vs 40.2 years; p<0,001), gender (69.2% males versus 76.4%; p<0.001) and insured capital (€273.116 vs €430.193; p<0.001). Thirty-two files were excluded, because of missing data for all relevant variables. The final sample used for the analyses included n = 15.094 files.

### Descriptives

Details of the characteristics of the study sample are shown in Table 1. The study sample consisted of n = 11.531 (76.4 percent) male applicants. Mean age was 40.2 year (SD 6.7 year). Of all applicants 25.9 percent disclosed they were smokers. Nearly 73.5 percent applied for ‘preferred life’. Ninety-five percent of all applicants were from higher socio-economic standard, based on criteria for income or asset. Also 39.8 percent fulfilled the criteria for a positive family history and 36.7 percent of the health declarations were estimated as leading to an extra-mortality of at least 25 percent due to any disclosed medical condition and/or use of blood pressure or lipids lowering drugs. On average the additional medical testing resulted in values within normal clinical limits.

### Associations between the separate independent variables and possible extra mortality

In the univariable analysis BMI and a positive SHD were significantly related to every single dependent variable. Gender, age and positive family history also showed significant associations although less strongly to every single dependent variable, except for age with cotinine. Table 2 shows an overview of all calculated areas under the curve (AUC) from univariable and multivariable regression analyses.

**Table 2:** Univariable and multivariable regression analyses calculated in area under the curve (AUC). Min. = minimum cut-off values. Max. = maximum cut-off values. Cum = cumulative. SBP = systolic blood pressure. DBP = diastolic blood pressure. TCHOL = total-cholesterol. TRI = triglycerides. Gluc = glucose. RAT = ratio total-cholesterol/HDL-cholesterol. COT = cotinine. BMI = body mass index. SHD = standard health declaration. FH = family history. Pref life = preferred life. SES = socio-economic status. All var = all independent variables. NA = not applicable.

	Treshold	BMI	Pos SHD	Gender	Age	FH	Smoking	Capital	PrefLife	SES	Model* Min	All var Min	Model** Max	All var Max.
**Overall**	Min.	0.680	0.591	0.586	0.576	0.530	0.512	0.520	0.522	0.504	0.708	0.710	0.741	0.743
**SBP**	Min.	0.675	0.646	0.578	0.596	0.532	0.509	0.495	0.522	0.500	0.729	0.730		0.798
**DBP**	Min.	0.700	0.645	0.570	0.583	0.539	0.513	0.523	0.525	0.498	0.740	0.740		0.842
**TCHOL**	Min.	0.625	0.606	0.547	0.556	0.540	0.510	0.505	0.512	0.504	0.658	0.664		0.732
**TRI**	Min.	0.678	0.561	0.593	0.562	0.525	0.512	0.523	0.523	0.500	0.696	0.696		0.888
**GLUC**	Min.	0.589	0.624	0.558	0.659	0.572	0.512	0.509	0.497	0.509	0.698	0.688		NA
**RAT**	Min.	0.721	0.633	0.594	0.591	0.540	0.510	0.513	0.538	0.497	0.759	0.757		NA
**COT**	Min.	0.590	0.603	0.576	0.531	0.505	0.650	0.500	0.507	0.502	0.733	0.751		NA

* Models for loading at minimum cut-off values. Model overall: BMI, positive health declaration and gender. Alternative model overall 1 = overall including family history 0.708. Alternative model 2 = alternative model 1 including age: 0.709. Model glucose: age, positive health declaration and family history. Alternative model glucose including BMI: 0.696. Model cotinine: smoking, gender and positive health declaration. Alternative model cotinine including BMI: 0.748.

** Models for loading at maximum cut-off values. Model Overall: BMI, positive health declaration and age. Alternative model = Overall including family history: 0.743.

A higher BMI was predominantly related to blood pressure and lipids. BMI showed an inverse relation with cotinine levels. Older age and a positive SHD were the dominating factors regarding glucose. Higher insured capital showed weak relations with diastolic blood pressure and triglycerides. Applying for preferred life rates showed weak associations with diastolic blood pressure, triglycerides and ratio total-cholesterol/HDL-cholesterol. The analyses did not show any other statistically significant relations between any of the independent and dependent variables.

### Outcome of the multivariable analyses

The value of the AUC for the overall dependent variable was 0.710 using the minimum cut-off levels for blood pressure and lipids and 0.743 using the maximum cut-off levels. Using a model including only those variables from the application form and the health declaration that were significantly associated with the outcome in the multivariable forward analysis model reduced the area under the curve to 0.708 at minimum cut-off levels and 0.741 at maximum cut-off levels. The multivariable forward analysis model for minimum cut-off levels included the BMI, the assessment of the SHD and gender. The model for maximum cut-off levels included the BMI, the assessment of the SHD and age.

## Discussion and Conclusion

### Main findings

In this sample of 15.094 life-insurance applicants we found that the BMI and the overall assessment of the health declaration were the dominant variables to discriminate between applicants for life-insurance with and without at least 25 percent extra mortality based on one or more of the dependent variables from the medical examination.

A higher BMI was predominantly related to blood pressure and lipids. This finding is in line with existing literature. Obesity class II and higher (BMI ≥ 35) has been shown to be associated with a significantly higher all-cause mortality [16,17]. The association between BMI and increased mortality due to vascular diseases is predominantly accounted for by blood pressure and lipids [18]. Extra-mortality due to BMI is age and gender dependent in the reinsurance manuals. Supported by literature [19] the risk calculators in guidelines of reinsurers manuals include the possible interaction between BMI, blood pressure and laboratory data. In addition BMI is associated with increased cancer risk as was shown in a recent large population-based cohort study [20].

Cancer and cardiovascular diseases are the main causes of death in both the general and insured population [21]. Medical underwriting in life insurance focuses on cardiovascular diseases and its risk factors like hypertension, elevated lipids and glucose levels as screening for cancer in a low risk population using imaging or advanced laboratory techniques is too costly and considered an unacceptable burden for the applicant. Relevant in this respect is the on average lower mortality risk in an insured population compared to the general population [22].

In the present study BMI was less clearly related to glucose. This can probably be explained by the fact that 90% of all diabetes is diabetes type II. Diabetes type II is more prevalent with increasing age [23,24]. Additionally, the association between BMI and diabetes type II is complex as patients with diabetes type II appear to vary greatly in pattern and to the degree of overweight at the time of diagnoses [25]. The disclosure of diabetes and diabetes-related disorders in the SHD may explain its association with elevated glucose level. These factors may account for the finding in our study that age and the outcome of the SHD instead of BMI were the dominant variables regarding a possible extra-mortality based on an elevated glucose level.

Gender, age and a positive family history were clearly less discriminating than BMI on the overall extra mortality as well as on most separate dependent variables. Age and gender had hardly any relation to blood pressure and lipids in multivariable analysis. This may reflect the age and gender dependency in the cut-off levels for blood pressure and lipids in the reinsurance manuals. Interaction between age, gender and blood pressure/lipids are in this way implicitly included in the outcome. Excluding the family history from the multivariable models hardly changed the results. Literature however shows a clear and consistent association between family history and premature cardiovascular morbidity and mortality, suggesting an inherited vulnerability [26,27]. The relative small impact of the family history in this study can only be explained by the notion that the BMI and a positive SHD explained a large part of the variation in outcome in the multivariable analyses.

Overall the non-medical variables from the application form showed no additional value in discriminating between persons that should additionally be medically tested. The amount of insured capital set by insurers as factor for additional medical testing could not be established in this study. This may contribute to the hypothesis that the amount of insured capital can be interpreted as a derivative of the socio-economic standard and related health behavior. Applicants asking for higher sum insured more likely belong to higher socio-economic classes, with subsequently lower mortality risks, than applicants applying for low sum assured as the sum assured in most cases will be linked to a mortgage or pension scheme. Relevant in this context is the possibility of adverse selection effects: the assumption that high risk applicants may be more likely to apply for insurance cover. However, applicants have to disclose all medical conditions known to them, irrespective of the sum assured, by the health declaration. Payment of the sum assured can be declined due to non-disclosure. A strong incentive for disclosing all relevant risk factors. According this study the sum assured is not a valid indicator for obligatory screening on medical risk factors unknown to the applicant. We assume insurers apply state-of-the-art health declarations and have professional underwriting and claim assessment at their disposal.

For three reasons models were used in order to calculate the loss in discriminative ability using limited variables compared to the model including all variables. First, law and legislation, for reasons of non-discrimination and accessibility, tend to get more and more restrictive on the underwriting process in private insurances. For instance the recent EU gender directive forces insurers to apply gender neutral premium rates [28]. Second, as for efficiency reasons insurers want to process as few data as possible. Third, to make the application process as client friendly as possible.

Using maximum instead of minimum cut-off levels in this study generated a relative small increase in the predicted probabilities. Due to higher cut-off levels the model will increase in discriminative power to select applicants without a possible extra-mortality.

### Strengths and weaknesses

The major strength of the present study is the size of the sample. In addition the medical examination data reflects an on average healthy population based on the screened risk factors blood pressure, lipids and glucose level. This matches with the on average low mortality risk in an insured population, being the target population. Weakness of the study is the fact we had to use register data. As a result we used the observed BMI, while the self-reported BMI would have ideally been used in our models. The self-reported BMI was however not available.

We missed medical data at lower sum assured as the level of medical guarantees are dependent of het level of the amount of insured capital set by the insurer. Therefore the outcome of this study is not applicable to applicants for life-insurance with low sums assured. A replication of the study among applicants with lower amounts of insured capital might be necessary. However in terms of costs, additional medical testing will be unattractive for insurers. In this respect the question is justifiable whether medical testing in cases of low insured capital compared to higher sum assured cases is worth considering.

### Practical and scientific implications

Implementation of a model with limited variables including BMI, SHD, age and gender can result in reducing the number of obligatory medical testing. This may reduce the costs of medical underwriting in life-insurance and at the same time speed-up the process and reduce the applicant burden. Studies comparing self-reported versus observed BMI show possible underestimation of the self-reported BMI at higher BMI levels [29]. Further research on self-reported versus observed BMI in the insured population is necessary. Furthermore, the significant differences in cut-off values of ordinary risk factors in reinsurance guidelines give rise to additional research on the evidence of these cut-off levels.

In this study we examined the prognostic value of parameters from the health declaration and application form on the probability of finding an assumed extra-mortality of at least 25%. Whether this increased probability results in an actual increase of (all cause) observed mortality compared to expected mortality gives rise to additional research.

In conclusion, results from this study indicate that BMI, the overall assessment of a health declaration and to a lesser extend age and gender, are the dominant discriminating variables to distinguish between applicants for standard term life-insurance with and without a possible minimal 25 percent extra mortality. The indication for additional medical testing at underwriting life-insurance can possibly be done on these limited variables instead of the obligatory medical testing based on age and the amount of insured capital. Prerequisites to prevent possible adverse selection effects is the use of a state-of-the-art health declaration and professional underwriting and claim assessment.

## Supporting Information

S1 Table Criteria preferred life. (DOCX)
