# Supplementary material for: The Added Value of Medical Testing in Underwriting Life Insurance
Source: PLoS One. 2015 Dec 30;10(12):e0145891. doi: 10.1371/journal.pone.0145891 (PMC4696800; doi:10.1371/journal.pone.0145891)
Supplement: S1 Table — (DOCX) [file pone.0145891.s001.docx]

**S1 Table. Criteria preferred life**

| **Variable** |  | **Age** |
| --- | --- | --- |
| BMI | ≥ 18 and ≤ 25  ≥ 18 and ≤ 26  ≥ 18 and ≤ 27 | 25-30 yr  31-50 yr  51-65 yr |
| Systolic blood pressure (mmHg) | ≥ 85 and < 130  ≥ 87 and < 135  ≥ 90 and < 140 | 25-40 yr  41-50 yr  51-65 yr |
| Diastolic blood pressure (mmHg) | ≥ 60 and < 90 |  |
| Total-cholesterol (mmol/L) | < 5.5  < 6.0 | 25-40 yr  41-65 yr |
| Ratio total-cholesterol/HDL-cholesterol | < 5 |  |
| Triglycerides (mmol/L) | < 4.5 |  |
| Calculatied LDL-cholesterol (mmol/L) | ≤ 4.1 |  |
| Random glucose (mmol/L) | < 7,8 |  |
| Family history | negative (1) |  |
| Smoking | non-smoker (2) |  |
| Health declaration | negative (3) |  |

| 1. no first degree relatives under age 50 with diabetes, cancer or cardiovascular disease, including CVA |  |
| --- | --- |
| 2. signed declaration of not smoking or using nicotine containing products in the past 24 months and a negative urine cotinin test | |
| 3. no antihypertension and/or lipids lowering drug use and no estimated extra-mortality of at least +25% due to any disclosed medical condition | |
